# The HIROSHIMA Study: A High-Volume, Institutional, Retrospective, Observational Study of Hidden Non-Incisional Suture Double Eyelid Surgery for Multivariate Analysis of Crease Loss

**DOI:** 10.1055/a-2796-6824

**Published:** 2026-03-27

**Authors:** Kyosuke Inoguchi, Takahiko Tamura, Hiromichi Okuma, Ryoichi Matsumoto, Michinori Yasuda, Takashi Yamauchi, Hiroo Teranishi

**Affiliations:** 1 Department of Aesthetic Surgery, Tokyo Chuo Beauty Clinic Fukuyama Clinic, Hiroshima, Japan; 2 Department of Aesthetic Surgery, Tokyo Chuo Beauty Clinic Umeda Osaka Ekimae Clinic, Osaka, Japan; 3 Department of Aesthetic Surgery, Tokyo Chuo Beauty Clinic Kobe Clinic, Hyogo, Japan; 4 Department of Aesthetic Surgery, Tokyo Chuo Beauty Clinic Okayama Clinic, Okayama, Japan; 5 Department of Aesthetic Surgery, Tokyo Chuo Beauty Clinic Hiroshima Clinic, Hiroshima, Japan

**Keywords:** non-incisional blepharoplasty, surgeon experience, eyelid surgery, crease loss, suture technique, aesthetic surgery, cosmetic, blepharoplasty, eyelid-double eyelid surgery-closed, eyelid

## Abstract

**Background:**

Non-incisional double eyelid surgery is widely performed in East Asia, but crease loss remains a major concern, leading to patient dissatisfaction and revision. The relative impact of surgical technique, anatomical characteristics, and surgeon experience on crease retention has not been fully clarified.

**Methods:**

This retrospective, observational study included 513 consecutive patients who underwent transcutaneous, non-incisional double eyelid surgery between July 2021 and July 2022. Patients were stratified by surgeon experience (<100 vs. ≥100 prior cases). Baseline variables included age, sex, crease design, fixation method, puffy eyelid status, and surgeon experience. Crease loss was defined as the need for revision due to patient-reported fading or disappearance of the crease. Kaplan–Meier analysis and Cox proportional hazards regression were used to evaluate predictors of crease survival, including interaction terms for potential effect modification.

**Results:**

Of 513 patients, 264 were treated by beginner surgeons, and 249 by experienced surgeons. Experienced surgeons more frequently used the continuous buried suture method ( *p* < 0.001), whereas baseline characteristics such as age, sex, and crease design were comparable between groups. Overall prevalence of puffy eyelids was 54.5%, with substantial interrater agreement (κ = 0.72). Kaplan–Meier analysis demonstrated significantly superior outcomes with continuous fixation compared to interrupted fixation (log-rank *p* < 0.001), and with non-puffy compared to puffy eyelids (log-rank *p* < 0.001). Surgeon experience showed no significant effect ( *p* = 0.441). In multivariable Cox regression, continuous fixation (HR = 0.29, 95% CI 0.19–0.44, *p* < 0.001) and non-puffy eyelids (HR = 2.47, 95% CI 1.62–3.78, *p* < 0.001) were independent predictors of crease retention. A significant interaction between fixation method and puffy eyelid was identified (HR = 0.34, 95% CI: 0.13–0.87, *p* = 0.024), indicating that the protective effect of continuous fixation was particularly pronounced in patients with puffy eyelids.

**Conclusion:**

Fixation method and eyelid puffiness were the primary determinants of crease retention, whereas surgeon experience was not an independent predictor. Continuous fixation provided superior outcomes, especially for patients with puffy eyelids. These findings suggest that, within standardized training systems, surgical technique and individualized anatomical consideration are more critical than case volume for achieving durable results in non-incisional double eyelid surgery.

## Introduction

Non-incisional double eyelid surgery is one of the most commonly performed aesthetic procedures in East Asia due to its minimally invasive nature, rapid recovery, and favorable cosmetic outcomes. 1 2 Despite its popularity, long-term crease stability remains a clinical challenge, with crease loss often leading to patient dissatisfaction and revision surgery. 3 Multiple factors, including fixation technique, eyelid anatomy, and surgeon experience, are presumed to influence outcomes, yet their relative contributions have not been clearly established. 4

Fixation methods are particularly important, with two major approaches widely used: Interrupted (tarsal-based) fixation and continuous (levator-based) fixation. Prior studies, including large-scale series, have suggested that continuous fixation may provide greater crease stability. However, many of these investigations were limited by retrospective designs, heterogeneous methodologies, or insufficient adjustment for confounding factors, such as surgeon experience and eyelid anatomy. 5 6 7 At the same time, anatomical characteristics such as puffy eyelids—caused by redundant skin, orbicularis hypertrophy, or orbital fat protrusion—are believed to compromise crease durability. However, these variables have rarely been systematically evaluated in large-scale analyses.

Surgeon experience is often assumed to be a key determinant of surgical success, particularly for procedures with a steep learning curve. Nonetheless, in the context of non-incisional double eyelid surgery performed within standardized training systems, the independent effect of experience on crease outcomes has not been rigorously quantified. Clarifying this relationship is critical not only for patient counseling but also for optimizing surgical education and protocol development in high-volume aesthetic practices.

To address these gaps, we conducted a large-scale, retrospective, observational study of 513 consecutive patients who underwent transcutaneous non-incisional double eyelid surgery in a standardized institutional setting. We aimed to evaluate the independent and interactive effects of fixation method, surgeon experience, and eyelid anatomy on crease retention, with particular attention to the role of puffy eyelids as a modifying factor.

## Methods

**Fig. 1:**
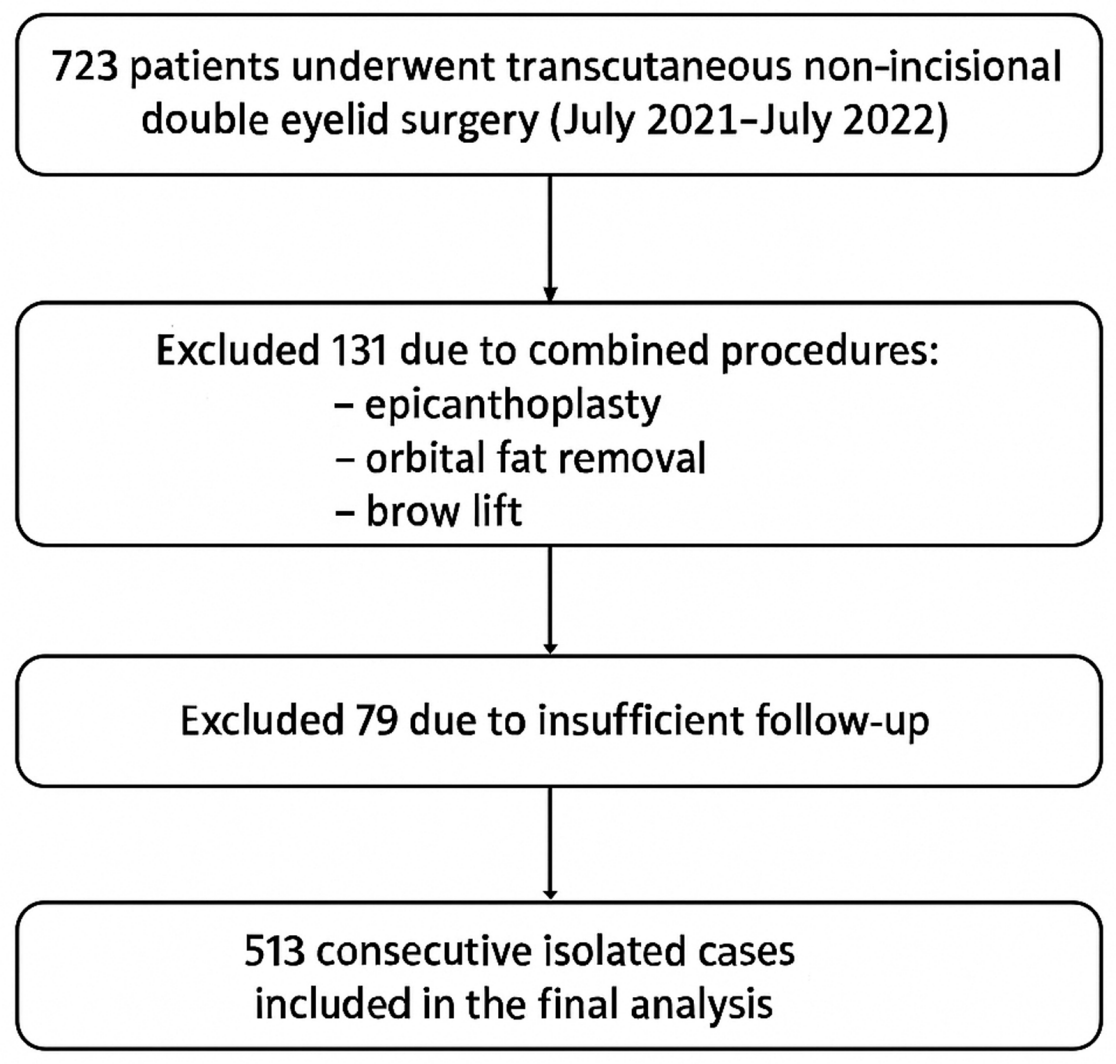
Flow chart of patient selection for the study. A total of 723 patients underwent transcutaneous, non-incisional double eyelid surgery between July 2021 and July 2022. Among them, 131 were excluded due to combined procedures (epicanthoplasty, orbital fat removal, or brow lift), and 79 were excluded due to insufficient follow-up, resulting in 513 consecutive isolated cases included in the final analysis.

This retrospective, observational study included patients who underwent transcutaneous non-incisional double eyelid surgery between July 2021 and July 2022 at a single institutional clinic belonging to a nationwide aesthetic surgery group of over 100 clinics. During the study period, 723 patients underwent the procedure. Of these, 131 were excluded due to combined procedures (epicanthoplasty, orbital fat removal, or brow lift), and 79 were excluded due to insufficient follow-up, resulting in 513 consecutive isolated cases for analysis. All patients were followed for 2 years postoperatively. Patient identification and procedure verification were confirmed using the institutional electronic surgical log and booking registry. The cohort selection process is illustrated in Fig. 1 . The study protocol was approved by the institutional ethics committee (approval number: UMEDAERB2025Jun010). Informed consent was obtained from the patient regarding the use of their photo. Patients were stratified according to the operating surgeon's level of experience: Those treated by surgeons with fewer than 100 prior cases were classified as the beginner group ( *n* = 264), and those treated by surgeons with 100 or more cases comprised the experienced group ( *n* = 249). The cutoff of 100 cases was adopted in accordance with precedents from surgical learning curve studies across various specialties. 8 9 10 Also, the cutoff of 100 cases was selected based on existing surgical learning curve literature across minimally invasive and aesthetic procedures, where proficiency typically stabilizes after 80 to 120 cases. The primary outcome was defined as crease loss, operationalized as the need for revision surgery due to patient-reported fading or disappearance of the previously formed crease.

**Fig. 2:**
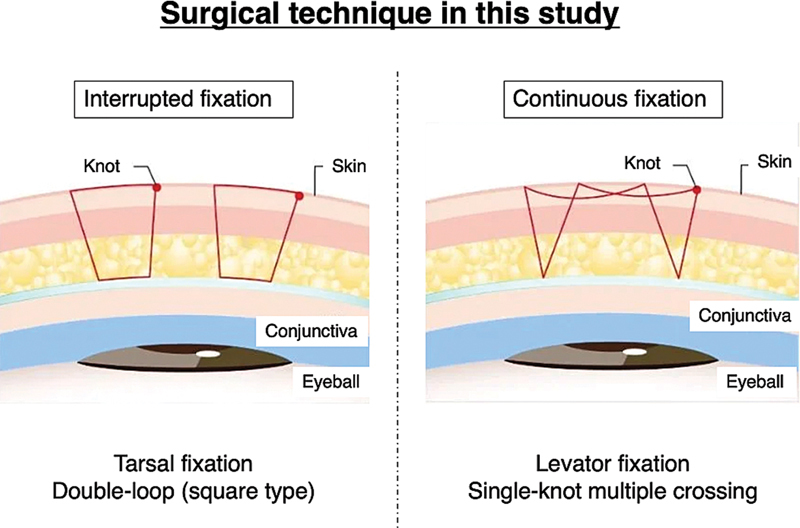
Schematic illustration of surgical fixation methods used in this study. (Left) *Interrupted fixation* : Tarsal fixation using a double-loop (square type) suture technique, with each loop tied separately to create multiple knots. (Right) *Continuous fixation* : Levator fixation using a single-knot, multiple-crossing suture technique, in which a continuous thread is passed across multiple points before being tied. Reproduced with permission from Tokyo Chuo Beauty Clinic.

**Fig. 3:**
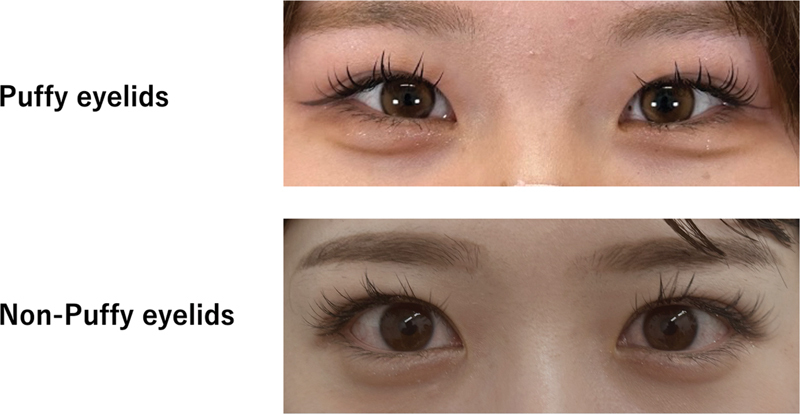
Representative preoperative clinical photographs illustrating eyelid morphology. Puffy eyelids were defined by prominent preaponeurotic fat resulting in a thick and edematous upper lid fold, whereas non-puffy eyelids exhibited minimal fat volume and a thinner appearance. These examples are provided to visually highlight the contrast between the two phenotypes.

Baseline variables included age, sex, eyelid crease design (tapered vs. parallel), fixation method, puffy eyelid status, and surgeon experience. Two fixation methods were employed ( Fig. 2 ). In the interrupted fixation technique (tarsal fixation, double-loop square type), independent sutures were placed to anchor the skin to the tarsal plate. In the continuous fixation technique (levator fixation, single-knot multiple crossing), a single thread was passed across multiple points between the skin and levator aponeurosis before being tied. Puffy eyelid was defined as anterior bulging of the upper eyelid contour caused by redundant skin, orbicularis muscle hypertrophy, thick retro-orbicularis oculi fat (ROOF), or orbital fat protrusion. In contrast, a *non-puffy eyelid* referred to an eyelid with minimal fat volume, where the upper lid skin appeared thin and less swollen. Representative clinical photographs are shown in Fig. 3 to illustrate the morphological differences between puffy and non-puffy eyelids. This was assessed using standardized preoperative frontal photographs, and two independent surgeons classified each case; discrepancies were resolved by consensus.

Statistical analyses were performed as follows. Categorical variables were compared using the chi-square test, and continuous variables with Student's *t* -test. Kaplan–Meier survival curves were generated to estimate crease retention, with time-to-event defined as the number of days from surgery to the date of confirmed crease loss. Group differences were assessed using the log-rank test. Cox proportional hazards regression was applied for univariate and multivariate analyses, reporting hazard ratios (HRs) with 95% confidence intervals (CIs). Interrupted fixation, non-puffy eyelid, beginner surgeon, and parallel crease design were set as the reference categories, with age analyzed as a continuous variable. The multivariable model included age, fixation method, surgeon experience, eyelid design, and eyelid puffiness, as well as interaction terms (puffy eyelid × method, operator × method, and operator × puffy eyelid) to evaluate potential effect modification. Statistical significance was set at *p* < 0.05.

In this study, model fit was evaluated using standard likelihood-based criteria, while detailed numerical indices such as the Akaike Information Criterion, log-likelihood values, and results from extended interaction models were relocated to the figure legends for clarity. All statistical analyses were performed using Python (version 3.12).

## Results

A total of 513 patients were included in the analysis. Baseline characteristics were largely comparable between groups. Beginner surgeons more frequently used the interrupted fixation method, whereas experienced surgeons predominantly employed the continuous method (χ ^2^ = 40.4, *p* < 0.001; Table 1 ). No significant differences were observed in sex distribution ( *p* = 0.602), eyelid crease design ( *p* = 0.089), or age (26.6 ± 11.7 years in beginners vs. 24.8 ± 10.1 years in experienced, *p* = 0.124). The prevalence of puffy eyelids was also similar between groups (60.6% vs. 48.2%, *p* = 0.091), with an overall rate of 54.5% across the cohort. Interrater agreement for puffy eyelid classification was substantial (κ = 0.72; agreement 85%).

**Table 1:** Baseline characteristics of patients stratified by surgeon experience (background characteristics)

Characteristic	Beginner ( *n* = 264)	Experienced ( *n* = 249)	*p* -Value
Age, mean ± SD (years)	26.6 ± 11.7	24.8 ± 10.1	0.124
Sex, *n* (%)			0.602
Male	35 (13.3)	29 (11.6)	
Female	229 (86.7)	220 (88.4)	
Crease design, *n* (%)			0.089
Parallel	172 (65.2)	142 (57.0)	
Tapered	92 (34.8)	107 (43.0)	
Fixation method, *n* (%)			<0.001
Interrupted	143 (54.2)	63 (25.3)	
Continuous	121 (45.8)	186 (74.7)	
Puffy eyelid, *n* (%)	160 (60.6)	120 (48.2)	0.091

The table presents the distribution of background characteristics among patients treated by experienced and beginner surgeons. Chi-square tests were used for categorical variables, and a two-sample *t* -test was applied for age.

**Fig. 4:**
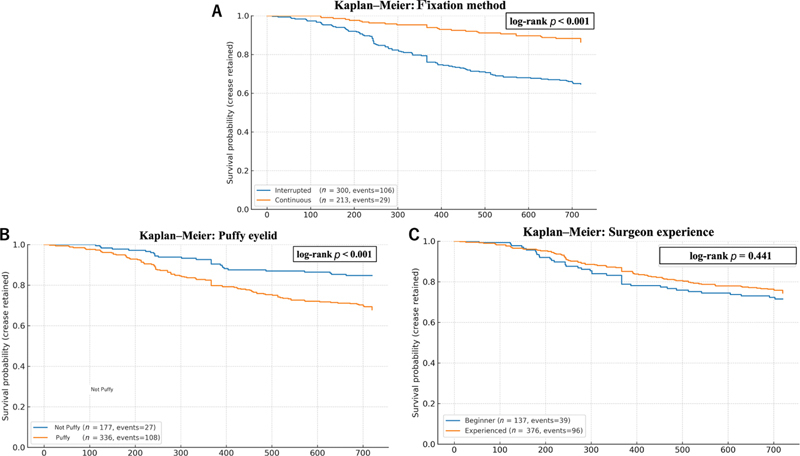
Kaplan–Meier survival curves **for** crease retention. ( **A** ) Kaplan–Meier survival curves stratified by fixation method (interrupted vs. continuous). The continuous method exhibited superior crease retention compared with the interrupted method (log-rank *p* < 0.001). ( **B** ) Kaplan–Meier survival curves stratified by eyelid type (puffy vs. non-puffy). Patients with puffy eyelids demonstrated significantly higher crease loss rates compared with non-puffy eyelids (log-rank *p* < 0.001). ( **C** ) Kaplan–Meier survival curves stratified by surgeon experience (beginner vs. experienced). No significant difference in crease retention was observed between groups (log-rank *p* = 0.441).

Kaplan–Meier analysis showed higher crease retention with the continuous fixation method than with the interrupted method (log-rank *p* < 0.001; Fig. 4A ). Similarly, non-puffy eyelids were associated with better long-term crease survival than puffy eyelids (log-rank *p* < 0.001; Fig. 4B ). In contrast, surgeon experience did not significantly affect crease retention (log-rank *p* = 0.441; Fig. 4C ).

**Fig. 5:**
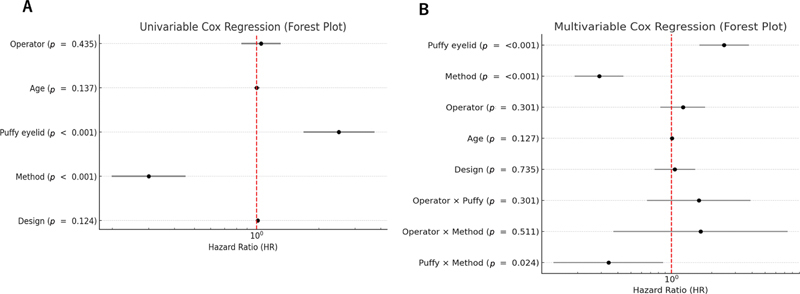
Cox regression analyses of factors associated with crease retention. ( **A** ) Univariable Cox regression forest plot showing hazard ratios for crease loss based on eyelid type (puffy vs. non-puffy), fixation method (interrupted vs. continuous), surgeon experience, and age. Both puffy eyelid and the interrupted fixation method were associated with a significantly increased risk of crease loss ( *p* < 0.001 for both). ( **B** ) Multivariable Cox regression forest plot including all covariates and interaction terms. Puffy eyelid (HR = 2.47, *p* < 0.001) and continuous fixation (HR = 0.29, *p* < 0.001) remained independent predictors of crease retention. The interaction term between puffy eyelid and fixation method was also significant (HR = 0.34, *p* = 0.024), suggesting that the negative effect of a puffy eyelid was partially mitigated when continuous fixation was used. In both panels, interrupted fixation, non-puffy eyelid, beginner surgeon experience, and parallel crease design were used as reference categories. Age was analyzed as a continuous variable.

In univariable regression, fixation method (HR = 0.33, 95% CI 0.22–0.49, *p* < 0.001) and eyelid puffiness (HR = 2.33, 95% CI 1.53–3.55, *p* < 0.001) were significant predictors of crease loss, whereas surgeon experience, eyelid design, and age were not significant ( Fig. 5A ; Table 2 ).

**Table 2:** Univariable and multivariable Cox regression analyses for predictors of crease loss

Variable	Univariable HR (95% CI)	*p* -Value	Multivariable HR (95% CI)	*p* -Value
Surgeon experience	0.86 (0.59–1.25)	0.435	1.22 (0.83–1.79)	0.301
Age	1.01 (0.99–1.03)	0.137	1.01 (0.99–1.03)	0.127
Puffy eyelid	2.33 (1.53–3.55)	<0.001	2.47 (1.62–3.78)	<0.001
Fixation method	0.33 (0.22–0.49)	<0.001	0.29 (0.19–0.44)	<0.001
Crease design	1.013 (0.996–1.030)	0.124	1.01 (0.96–1.07)	0.735
Operator × Puffy	–	–	0.83 (0.52–1.34)	0.301
Operator × Method	–	–	0.89 (0.62–1.29)	0.511
Puffy × Method	–	–	0.34 (0.13–0.87)	0.024

Abbreviations: CI, confidence interval; HR, hazard ratio.

HRs with 95% CIs and *p* -values are presented. Variables with *p* < 0.05 were considered statistically significant. In multivariable analysis, puffy eyelid and fixation method remained independent predictors of crease loss, and a significant interaction was observed between puffy eyelid and fixation method.

In the multivariable Cox regression model, both puffy eyelid (HR = 2.47, 95% CI: 1.62–3.78, *p* < 0.001) and fixation method (HR = 0.29, 95% CI: 0.19–0.44, *p* < 0.001) remained significant predictors ( Fig. 5B ).

Surgeon experience (HR = 1.22, 95% CI: 0.83–1.79, *p* = 0.301) and age (HR = 1.01, 95% CI: 0.99–1.03, *p* = 0.127) were not significant after adjustment. Importantly, an interaction was observed between puffy eyelid and fixation method (HR = 0.34, 95% CI: 0.13–0.87, *p* = 0.024), indicating that continuous fixation was especially protective in puffy eyelids. No significant interactions were identified between surgeon experience and fixation method ( *p* = 0.511) or between surgeon experience and puffy eyelid ( *p* = 0.301). The multivariable model demonstrated good fit, and the inclusion of interaction terms did not alter the overall results or reach statistical significance. Related model fit indices and supplementary interaction analyses are summarized in Fig. 5A, B .

## Discussion

This study examined the effects of surgeon experience, fixation method, and eyelid characteristics on crease loss following transcutaneous, non-incisional double eyelid surgery. Fixation method and puffy eyelid status were the primary determinants of crease retention, whereas surgeon experience was not an independent factor after adjustment. Age and eyelid design also showed no significant associations.

Puffy eyelid was objectively defined from standardized preoperative photographs, with substantial interrater agreement (κ = 0.72), supporting the reliability of this classification. While more advanced imaging could further refine anatomical assessment, our binary classification provided a practical framework for large retrospective datasets. A 100-case cutoff was selected based on prior learning-curve studies. 8 9 10

A significant imbalance in fixation technique usage was observed: Experienced surgeons more frequently selected the continuous buried suture method. This likely reflects accumulated familiarity or preference. Although experienced surgeons more frequently used continuous fixation, surgeon experience showed no independent association with crease loss after adjustment for fixation method. Therefore, the apparent effect of experience may be mediated through technique choice rather than inherent operator skill. Prior reports similarly support the superiority of continuous fixation in crease longevity. 4 5 6 7

Our analysis further demonstrated that the protective effect of continuous fixation was particularly evident in patients with puffy eyelids, indicating an important method–anatomy interaction. By contrast, surgeon experience was not a significant predictor in either Kaplan–Meier or Cox regression analyses, nor did it modify the effect of fixation method. These findings suggest that, within a standardized educational environment, novice surgeons can achieve outcomes comparable to those of experienced surgeons when appropriate techniques are applied.

Overall, crease retention depended more on fixation method and eyelid anatomy than on surgeon experience. Continuous fixation yielded superior outcomes compared with interrupted fixation, while patients with puffy eyelids faced a markedly higher risk of crease loss. Importantly, the statistically significant interaction between puffy eyelid and fixation method highlights the need for individualized technique selection, especially in high-risk patients.

This study has several limitations.

1. Puffy eyelid status was assessed retrospectively from preoperative photographs, which may introduce misclassification bias despite substantial interrater agreement.
2. Photographic conditions were not fully standardized, potentially affecting the consistency of crease evaluation across patients.
3. As a retrospective, observational study, the dataset lacked control over certain variables, and data granularity was limited by available clinical records.
4. The study was conducted at a single institution, which may limit generalizability to other populations or surgical settings.
5. Future multicenter, prospective studies with quantitative crease measurements and standardized imaging are warranted to validate these findings.

In conclusion, the fixation method emerged as the most important predictor of crease retention, while surgeon experience did not independently influence outcomes after adjustment. These findings suggest that the non-incisional technique, when performed with an appropriate fixation method, can yield consistent and durable results even in the hands of less experienced surgeons. While standardized training may facilitate dissemination of effective methods, our results indicate that surgical technique itself, rather than surgeon experience or training volume, is the major determinant of long-term crease survival.
